# Photon-phonon Interaction in a Microfiber Induced by Optical and Electrostrictive Forces

**DOI:** 10.1038/srep41849

**Published:** 2017-02-01

**Authors:** Yun-chao Shi, Wei Luo, Fei Xu, Yan-qing Lu

**Affiliations:** 1 National Laboratory of Solid State Microstructures, and College of Engineering and Applied Sciences, Nanjing University, Nanjing 210093, People’s Republic of China

## Abstract

Stimulated Brillouin scattering (SBS) via electrostrictive force is a fundamental interaction between light and sound which limits the power in conventional optical fibers. The emergence of optical microfibers with subwavelength diameter, ultralight mass and an intense light field, provides a new platform for photon–phonon coupling, resulting in the radiation pressure mediated contribution of SBS. This study examines the optomechanical system in cylindrical coordinates, reveals the theoretically radiation pressure induced analogous, and demonstrates contrary effect compared with electrostrictive force in solid or hollow silica microfibers. The finding shows that the photon-phonon coupling, which is related to SBS, can be suppressed in a solid microfiber, and even be completely cancelled in a hollow microfiber.

Photon-phonon coupling in nanoscale waveguides through guided-wave stimulated Brillouin scattering (SBS) has recently emerged as an important area of research. In suspended rectangle silicon waveguides, photon-phonon coupling has been considered as a result of a coherent combination of electrostrictive forces and boundary-induced radiation pressures^1^,^2^,^3^,^4^,^5^. The enhanced Brillouin nonlinearities caused by the emergence of large radiation pressure-induced couplings provides a means of nonlinear signal processing. In conventional cylindered fibers, however, it is difficult to observe and utilize analogous optomechanical responses due to the fiber size and device configuration. Brillouin nonlinearity is main form of electrostrictive forces and has important applications ranging from optical fiber sensors to optical memory. In most cases, Brillouin nonlinearity is harmful because it is the major factor limiting the power of a fiber device. There are several methods to suppress it, e.g. broadening the effective Brillouin gain bandwidth^6^, using a nonuniform fiber which has a large core^7^, using a different fiber structure or changing fiber parameters^8^.

Recently, thanks to the enormous progress in the fabrication of low-loss submicrometric optical wires, optical microfibers have attracted more attention because of various eminent advantages: low cost, tremendously large evanescent field, light weight, and low-loss interconnection to standard fiber devices. The core of the microfiber can be both solid and hollow. The situation for photon–phonon coupling changes dramatically in microfibers in consideration of their extreme light weight and free-standing status, which gives rise to radiation pressure mediated contribution of SBS. In particular, the new contribution to SBS coupling is possibly negative because of the unique cylindrical geometry. It will provide an alternative mechanism to suppress SBS in fiber based micro-devices^9^,^10^,^11^. A possible reason is that the responses of microfiber’s refractive index caused by both electrostrictive forces and radiation pressures have the same order of magnitude. They can be counteracted in cylindrical geometry due to high symmetries.

In this paper, we analyse the radiation pressure and electrostrictive force, which could drive phonon creation and cause SBS^12^,^13^,^14^,^15^ in microfibers, showing that the radiation pressure and electrostrictive force are comparable. These two forces are in opposite directions: the electrostrictive force tends to compress the microfiber while radiation pressure tends to extend the microfiber. The electrostrictive force is always larger than radiation pressure in a solid microfiber. Then, we change the structure of microfiber and analyse the hollow microfiber. In the hollow microfiber, the electrostrictive force can completely counteract radiation pressure at a certain size. The radiation pressure is larger than the electrostrictive force at a small size, which means the effect of the electrostrictive force can be counteracted by adjusting the size and structure of the microfiber. The schematic in Fig. 1 illustrates optical and acoustic modes profiles for the solid microfiber and the hollow microfiber, which are computed by numerical simulation. Furthermore, through adjusting the size and structure of microfibers, Kerr effect can be enhanced or decreased by elasto-optical effect, which is caused by the radiation pressure and electrostrictive force.

The strength of SBS is characterized by the SBS gain which scales quadratically with the overlap of photon-phonon interaction^1^,^2^,^16^,^17^,^18^. The calculation shows that the photon-phonon coupling can be weakened in the solid microfiber. In the hollow microfiber, the photon-phonon coupling can even be completely cancelled. This means the loss in the microfiber can be reduced through decreasing the SBS gain. These results thus show the potential of optical microfiber for low-threshold lasers and amplifiers.

## Results and Discussion

### Analysis of radiation pressure and electrostrictive force in microfiber

The radiation pressure (electrostrictive forces) in the dielectric media can be computed using Maxwell stress tensor (electrostrictive stress) (see Methods). A suspended solid microfiber is assumed to be less than 1 μm in radius. The optical mode traveling in the solid microfiber is the fundamental mode. It’s easier to analyse the radiation pressure and electrostrictive force using cylindrical coordinate system in a solid microfiber.

In a solid microfiber, we analyse the radial force. Through the transformation of Maxwell stress tensor from Cartesian coordinate system to the cylindrical coordinate system, the radial component of Maxwell stress tensor is:


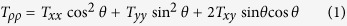


here, 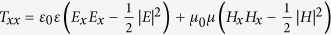, 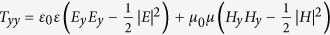 and 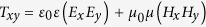.

The electrostrictive stress is s a fourth-order tensor and it can be expressed in contracted notation due to symmetry. Amorphous material has high symmetry (photoelastic coefficients *P*_*13*_ = *P*_*12*_). The photoelastic coefficients of silica are: *P*_*11*_ = 0.121, *P*_*12*_ = 0.27, *P*_*44*_ = −0.075^19^, so the radial component of electrostrictive stress is analogous:


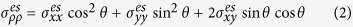


here, 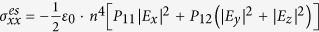 and 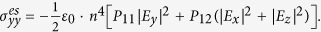



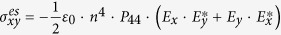


n is the refractive index. We find that 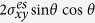 can be omitted in the next calculations.

To compare the radiation pressure with the electrostrictive forces in a solid microfiber, the method mentioned in the literature^20^ is used to estimate the aggregate forces which act to deform a solid microfiber, we define the spatial averaged stress:


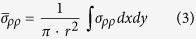


We compute the aggregate force through virtual work formulation:


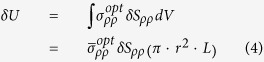


here, *δU* is the change in total energy, 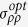 represents 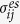 or 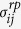 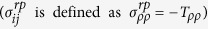, *δS*_*ρρ*_ = *δr*/*r, δr* is a virtual displacement in radial direction. *L* is the length of solid microfiber. *P* is the power in a solid microfiber.

The aggregate force (power normalized force per unit length) is:


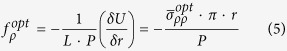


Through the analyses above, the radiation pressure is:


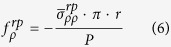


The electrostrictive force is:


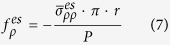


We can compute the force with the radius of a solid microfiber varying from 0.2 μm to 1 μm, and the result is shown in Fig. 2(a).

From Fig. 2(a), the sign is different between the radiation pressure and the electrostrictive force, which means that radiation pressure expands the solid microfiber while electrostrictive force compresses it. Because the photoelastic coefficients of silica are positive, the electrostrictive force is negative from Eq. (10). No matter how the radius changes in the range, electrostrictive force is stronger than radiation pressure. So the total force that is negative compresses the solid microfiber. When the diameter is around 1 μm, radiation pressure and electrostrictive force reach the maximum. If we can find materials whose photoelastic coefficients are negative, both of the force will be in the same direction. In this manner, we can enhance the force using this material.

Further, we try to counteract the radiation pressure with the electrostrictive force, because the magnitudes of both of the forces are close. We analyse a hollow microfiber instead of a solid microfiber. The radius of the hollow core is fixed at 0.2 μm, the thickness of silica ring varies from 0.1 μm to 1 μm. Using the same method, the expression for both of the power normalized force per unit length is:


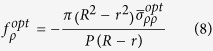


here, 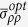 represents spatial averaged stress of radiation pressure or electrostrictive force, *R* is the radius of the hollow microfiber, *r* is the radius of hollow core and *P* is the power in the hollow microfiber.

We calculate radiation pressure and electrostrictive force and show them in Fig. 2(b). When the silica ring is thin, the radiation pressure is stronger than electrostrictive force. This is different from the situation in a solid microfiber, the reason being that optical field distribution concentrates in the center of a solid microfiber while optical field distribution is closer to boundaries in a hollow microfiber. Radiation pressure counteracts electrostrictive force when the thickness of the silica ring is around 0.75 μm. This means that the effect of electrostrictive force can be completely cancelled at this size. The intuitive images about radiation pressure optical and electrostrictive force are shown in Fig. 2(c).

To verify it, we analyse the second-order mode in a solid microfiber whose radius is below 2 μm. Figure 3 shows that the radiation pressure is larger than electrostrictive force. Both of the forces reach the maximum value when the radius is 0.7 μm. In this case, the peak value of the radiation pressure almost reaches 10 pN/μm/mW which is larger than that we discussed in the solid microfiber and the hollow microfiber. The result that the radiation pressure holds a dominant position is contrary to the situation in the fundamental mode.

### Analysis of photon-phonon interaction in microfibers

Through changing the structure of a microfiber, the sign of aggregate force varies from positive to negative. The electrostrictive force dominates in compressing the solid microfiber. In a hollow microfiber, the radiation pressure holds a dominant position when the thickness of silica ring is small.

As is well-known, the phonon is driven by electrostriction in the process of SBS in microfibers^2^. We can counteract the electrostrictive force with radiation pressure. So, SBS can be weakened or even completely inhibited in microfibers. First, we consider a solid microfiber suspended in air considering the fundamental mode. The Brillouin parameter which is related to photon-phonon interaction is ref. 3:


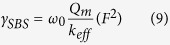


here, 2*γ*_*SBS*_ = *G*_*SBS*_(Ω_*m*_) is the Brillouin gain coefficient, *Q*_*m*_ is a quality factor, *ω*_0_ is the frequency of the pump, *k*_*eff*_ is the effective stiffness coefficients, 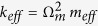, *m*_*eff*_ is the effective mass of the mechanical mode per unit length and Ω_*m*_ is the phonon resonance frequency, *F* is radiation pressure or electrostrictive force (power normalized force per unit length). Substituting parameters of a solid microfiber and simplifying:


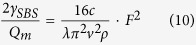


here, *c* is velocity of light, *λ* is the wavelength of the pump, *v* is the longitudinal speed of sound in silica and *ρ* is density of silica.

We have computed both of the forces in a solid microfiber, so 2*γ*_*SBS*_/*Q*_*m*_ in a solid microfiber is shown in Fig. 4(a). 2*γ*_*SBS*_/*Q*_*m*_ is a direct measure of the photon-phonon overlap in the non-resonant part. Because the electrostrictive force and radiation pressure are in contrary directions, the two contributions weaken the photon–phonon coupling strength. The total photon-phonon overlap is about one fifth of that contributions by electrostriction. To completely cancel the photon-phonon coupling, we consider the situation in a hollow microfiber. The radius of the hollow core is fixed at 0.2 μm, the thickness of silica ring varies from 0.1 um to 1 um. Using the force we have computed, we can see the result in Fig. 4(b). The photon-phonon coupling strength becomes zero because radiation pressure counteracts electrostrictive force while the thickness of silica ring is around 0.8 μm. This means we completely cancel the Brillouin gain in the hollow microfiber.

## Conclusions

In this work, we theoretically demonstrate that the direction of the aggregate force is dependent on the structure of microfibers. In a solid microfiber, the electrostrictive force is stronger than the radiation pressure, compressing the solid microfiber. In a hollow microfiber, the radiation pressure is stronger than the electrostrictive force when the radius is within 0.8 μm, extending the hollow microfiber. The effect of electrostrictive force can be completely counteracted by radiation pressure when the thickness is 0.8 μm. Thus, different forces hold a dominant position in different systems. Next, we analyze SBS gain, considering the effect of both radiation pressure and electrostrictive force. Our calculation results show that SBS gain can be weakened in a solid microfiber or even completely inhibited in a hollow microfiber. This discovery can enhance channel power in optical communication system because SBS can be dramatically decreased in microfibers by changing the structure of microfibers.

## Method

### Numerical simulation

**Figure 1:**
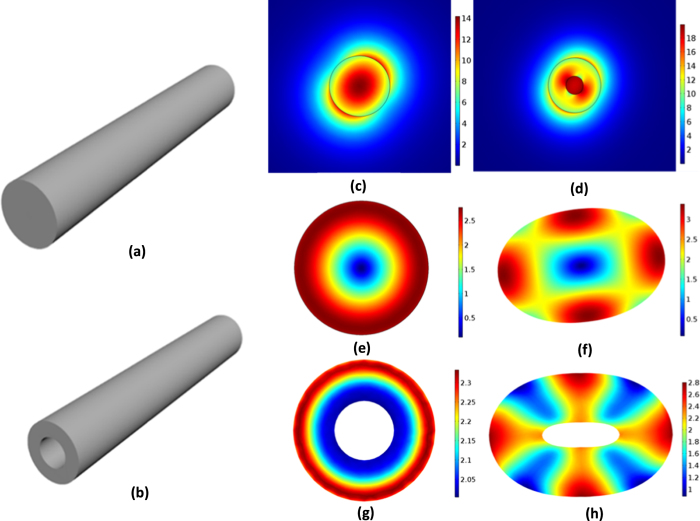
Optical and acoustic modes profiles for the solid microfiber and the hollow microfiber. The diameter of the solid microfiber is 1 μm. The inner and outside diameters of the hollow microfiber is 0.4 μm and 1.2 μm. The schematic diagram of the solid microfiber in (**a**) and the hollow microfiber in (**b**). Electric field norm in the fundamental optical mode of the solid microfiber in (**c**) and the hollow microfiber in (**d**). Two acoustic modes involved in stimulated brillouin scattering: axially asymmetric torsional-radial mode of the solid microfiber in (**f**) and the hollow microfiber in (**h**), axially symmetric radial mode of the solid microfiber in (**e**) and the hollow microfiber in (**g**).

**Figure 2:**
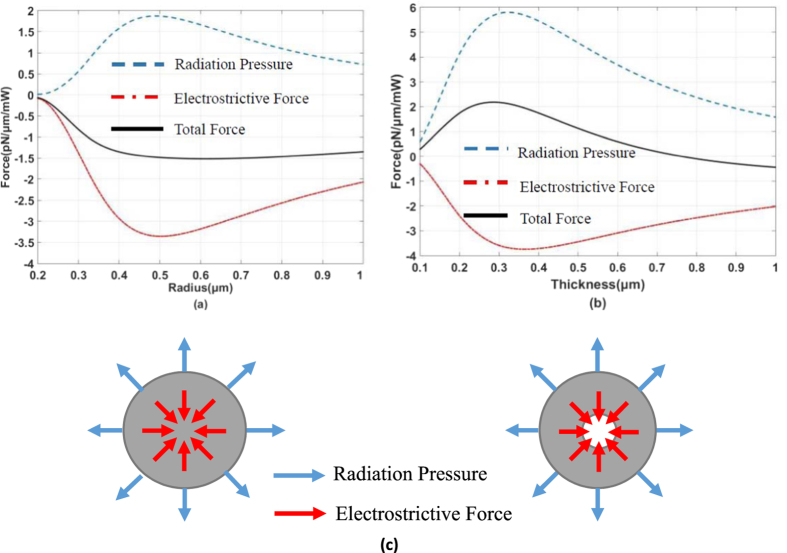
Relation of the force and radius. (**a**) The force is calculated in solid microfibers. The force is normalized by optical power. (**b**) The force is calculated in hollow microfibers. Radiation pressure counteracts electrostrictive force while the thickness of silica ring is around 0.75 μm. (**c**) Illustration of the radiation pressure and electrostrictive force. In both of a solid microfiber and a hollow microfiber, radiation pressure expands the microfiber while electrostrictive force compresses it.

**Figure 3:**
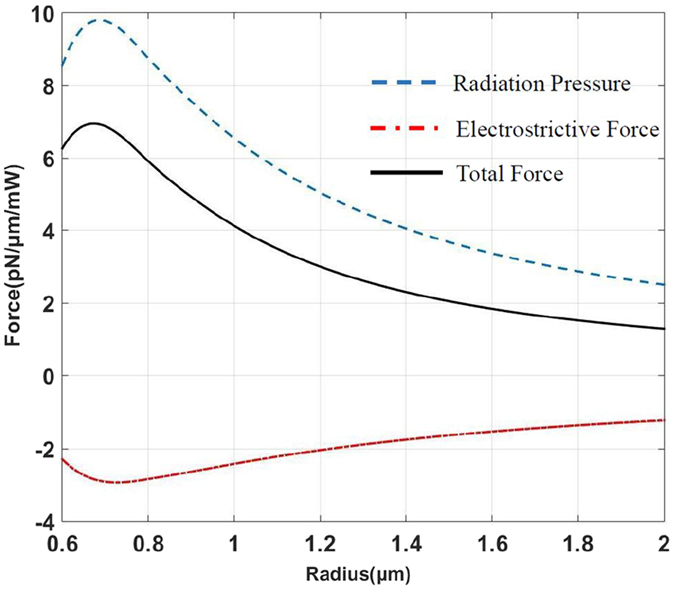
Relation of the force and radius. The force is calculated in the second-order mode in a solid microfiber by using Eq. (10). The force is normalized to optical power. The result is totally different from that in the fundamental mode. Radiation pressure is larger in the second-order mode instead of electrostrictive force.

**Figure 4:**
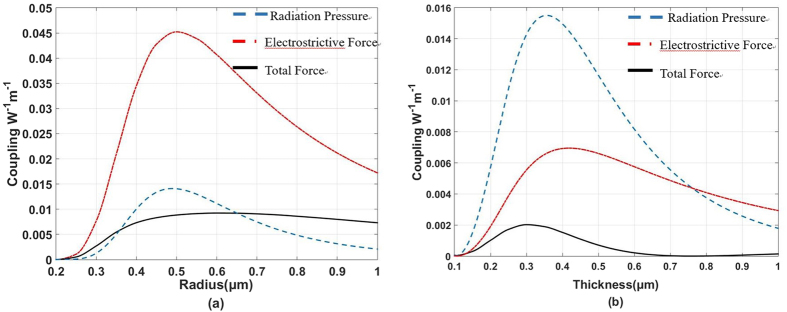
Illustration of the photon-phonon overlap. Different lines represent different situation. For example, blue line represents that the photon-phonon overlap is calculated by only considering the influence of radiation pressure. (**a**) In a solid microfiber, the photon-phonon overlap caused by total force is smaller than that caused by the electrostrictive force. (**b**) In a hollow microfiber, the photon-phonon overlap caused by total force can be counteracted while the thickness of silica ring is around 0.8 μm.

Our numerical simulations are based on numerical simulation using finite element method (COMSOL Multiphysics). The results in Fig. 1(c) and (d) are simulated by RF module of COMSOL, and the results in Fig. 1(e) and (h) are simulated by structural mechanics module of COMSOL.

### General method of calculating radiation pressure and electrostrictive force

The radiation pressure in the dielectric media can be computed using Maxwell stress tensor. The formula is ref. 21:


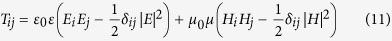


where *ε*_0_(*μ*_0_) is the electric permittivity (magnetic permeability) in free space, *ε(μ*) is the relative electric permittivity (magnetic permeability). *E*_*i*_, *E*_*j*_ is the electric (magnetic) field component. *δij* is Kronecker sign function. Similarly, electrostrictive forces can be computed through electrostrictive stress^20^:


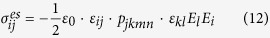


where *p*_*jkmn*_ is the photoelastic tensor.

We obtained the photonic and phononic modes from the finite-element solver COMSOL. The results were exported to MATLAB to calculate 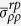 and  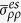 (defined by Eq. (3)). Then 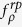 and 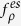 can be calculated by Eqs (6) and (7).

With the forces above, we analyzed photon-phonon interaction in microfibers, 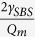 can be calculated by Eq. (10)

Here, we derived Eq. (10)


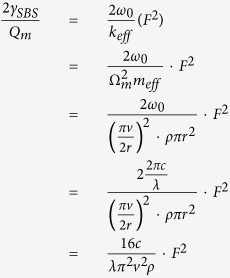


Here, 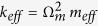, *m*_*eff*_ is the effective mass of the mechanical mode per unit length, Ω_*m*_ is the phonon resonance frequency, *c* is velocity of light, *λ* is the wavelength of the pump, *v* is the longitudinal speed of sound in silica and *ρ* is density of silica.

## Additional Information

**How to cite this article**: Shi, Y.-c. *et al*. Photon-phonon Interaction in a Microfiber Induced by Optical and Electrostrictive Forces. *Sci. Rep.* **7**, 41849; doi: 10.1038/srep41849 (2017).

**Publisher's note:** Springer Nature remains neutral with regard to jurisdictional claims in published maps and institutional affiliations.
