# Corrigendum: Photon-phonon Interaction in a Microfiber Induced by Optical and Electrostrictive Forces

**DOI:** 10.1038/srep46862

**Published:** 2017-07-10

**Authors:** Yun-chao Shi, Wei Luo, Fei Xu, Yan-qing Lu

Scientific Reports 7: Article number: 41849; 10.1038/srep41849 published online: 02 01 2017; updated: 07 10 2017.

Experimental and theoretical results of spontaneous Brillouin scattering (SpBS) in solid silica microfibers have been demonstrated^1^,^2^. Surface acoustic waves and Brillouin scattering self-cancellation were found and discussed in the two papers^1^,^2^. In our paper, we investigated the stimulated Brillouin scattering (SBS) process in solid and hollow microfibers. Our conclusion was that SBS can be suppressed in a solid microfiber and even be completely cancelled in a hollow microfiber. Our theoretical model did not include SpBS which originates from the acoustic waves caused by Brownian motion (thermal motion), thus self-cancellation effect of SpBS in solid microfiber was not demonstrated in our work.
